# Multidrug Resistance in Enterococci Isolated From Wild Pampas Foxes (*Lycalopex gymnocercus*) and Geoffroy's Cats (*Leopardus geoffroyi*) in the Brazilian Pampa Biome

**DOI:** 10.3389/fvets.2020.606377

**Published:** 2020-12-04

**Authors:** Gabriella Oliveira de Araujo, Rosana Huff, Marina Ochoa Favarini, Michele Bertoni Mann, Felipe Bortolotto Peters, Jeverson Frazzon, Ana Paula Guedes Frazzon

**Affiliations:** ^1^Graduate Program in Agricultural and Environmental Microbiology, Institute of Basic Health Sciences, Federal University of Rio Grande Do Sul, Porto Alegre, Brazil; ^2^Institute for the Conservation of Neotropical Carnivores— “Pró-Carnívoros”, Atibaia, Brazil; ^3^Institute of Food Science and Technology, Federal University of Rio Grande Do Sul, Porto Alegre, Brazil

**Keywords:** *Enterococcus* spp., pampa biome, wildlife animals, Pampas fox, Geoffroy's cat, multidrug-resistance, virulence factors, antibiotic resistance genes

## Abstract

Enterococci are ubiquitous microorganisms present in various environments and within the gastrointestinal tracts of humans and other animals. Notably, fecal enterococci are suitable indicators for monitoring antimicrobial resistance dissemination. Resistant bacterial strains recovered from the fecal samples of wild animals can highlight important aspects of environmental disturbances. In this report, we investigated antimicrobial susceptibility as well as resistance and virulence genes in fecal enterococci isolated from wild Pampas foxes (*Lycalopex gymnocercus*) (*n* = 5) and Geoffroy's cats (*Leopardus geoffroyi*) (*n* = 4) in the Brazilian Pampa biome. Enterococci were isolated from eight out of nine fecal samples and *Enterococcus faecalis* was identified in both animals. However, *E. faecium* and *E. durans* were only detected in Pampas foxes, while *E. hirae* was only detected in Geoffroy's cats. Antimicrobial susceptibility analysis showed resistance to rifampicin (94%), erythromycin (72.6%), ciprofloxacin/norfloxacin (40%), streptomycin (38%), and tetracycline (26%). The high frequency of multidrug-resistant enterococci (66%) isolated in this study is a matter of concern since these are wild animals with no history of therapeutic antibiotic exposure. The *tet*M/*tet*L and *msr*C/*erm*B genes were detected in most tetracycline- and erythromycin-resistant enterococci, respectively. The *gelE, ace, agg, esp*, and *clyA* virulence genes were also detected in enterococci. In conclusion, our data suggest that habitat fragmentation and anthropogenic activities in the Pampa biome may contribute to high frequencies of multidrug-resistant enterococci in the gut communities of wild Pampas foxes and Geoffroy's cats. To the best of the authors' knowledge, this is the first report of antimicrobial-resistant enterococci in the Pampa biome.

## Introduction

Brazil hosts six terrestrial biomes, which include the Amazon, Atlantic Forest, Caatinga, Cerrado, Pampa, and Pantanal biomes. Notably, the Pampa biome covers 63% of Rio Grande do Sul State and extend to Uruguay and the central region of Argentina (1–3). The fauna of the Brazilian Pampa biome consists of 83 native mammal species, of which some are endemic and/or considered endangered species. Among the mammal species, Geoffroy's cat (*Leopardus geoffroyi*) (Felidae) and the Pampas fox (*Lycalopex gymnocercus*) (Canidae) are listed as species of “least concern” in the IUCN Red List of Threatened Species (4, 5). The main factors contributing to the decline of these species are habitat destruction and hunting (2, 6, 7). Farming activities have converted natural areas of the Brazilian Pampa into agricultural and grazing lands, with ~48.7% of this biome now being used for plantation crops (1, 3).

This biome has been suffering constant disturbances due to anthropogenic impacts and the reduction of natural habitat has forced wild animals to live near human settlements, which has resulted in negative outcomes for wildlife conservation (8, 9). Pampas fox and Geoffroy's cat population density in Brazilian Pampa biome is 0.2 and 0.27 ind/km^2^, respectively (10, 11). Studies of wild canids and felids from the Pampa biome have shown that these animals exhibit adaptability in foraging based on prey availability, which can lead them to establish secondary food sources on farms. They are known to consume domestic vertebrates, fruit, insects, and carrion as well as to get food into the farms trash (12–14). In the past year, various studies have been published regarding habitat degradation and its effects on the wildlife and environment of the Pampa biome; however, studies evaluating the impact of multidrug-resistant bacteria on the wildlife in this biome remain scarce.

Enterococci are ubiquitous microorganisms found in water, soil, plants, and gastrointestinal tracts of wild animals, domestic animals, and humans (15–19). This ubiquitous distribution has been associated with phenotypic plasticity since they can tolerate a wide range of temperature and pH and grow in the presence of 6.5% sodium chloride (NaCl) or 40% of bile salts (20). The genus *Enterococcus* comprises at least 50 species (21). Among these, *E. faecalis* is the predominant species in the gastrointestinal tracts of mammals, followed by *E. faecium, E. durans, E. hirae*, and *E. mundtii* (18).

Additionally, enterococci are considered opportunistic pathogens in susceptible hosts. They cause urinary tract, wound, and soft tissue infections as well as bacteremia (22, 23). Although enterococci are considered a common cause of nosocomial infections, they can also cause several diseases including bovine mastitis, endocarditis, septicemia, and diarrhea in dogs, cats, pigs, and rats (24). The treatment of enterococcal infections has been complicated by the emergence of antibiotic-resistant strains, which makes these infections an important public health concern. Resistance to different classes of antimicrobials is a hallmark of *Enterococcus* spp. since they are intrinsically resistant to β-lactams, cephalosporin, lincosamides, streptogramins, and aminoglycosides (25). Meanwhile, resistant strains are not restricted to clinically known species since such strains have been isolated from different environments, including wildlife (15, 17, 19, 24, 26–30). Due to their remarkable ability to adapt to the environment, ubiquity in gut and to acquire antibiotic resistance determinants, enterococci have been employed as sentinel organisms for resistance to antimicrobials with Gram-positive activity.

Resistant bacterial strains recovered from wild animals can highlight important aspects of microbial interactions and environmental disturbances in wildlife (31, 32). Wild animals can be considered sentinels for the emergence and spread of antimicrobial-resistant bacteria in the environment. Therefore, the present study evaluated the presence of resistant enterococci in wild mammals aiming to detect previously unstudied variation in antimicrobial resistance distribution patterns in these animals. Additionally, to date, relatively few reports on antimicrobial resistance strains have been produced based on samples from wild canids and felids when compared to the number of reports on domestic animals. This difference could largely be explained by the migratory habits of some wild species and the difficulty of obtaining samples from wildlife. To the best of the authors' knowledge, this is the first study of antimicrobial resistance profiles and virulence genes in fecal enterococci isolated from wild Pampas foxes and Geoffroy's cats in the Brazilian Pampa biome.

## Materials and Methods

### Samples Collection

**Figure 1:**
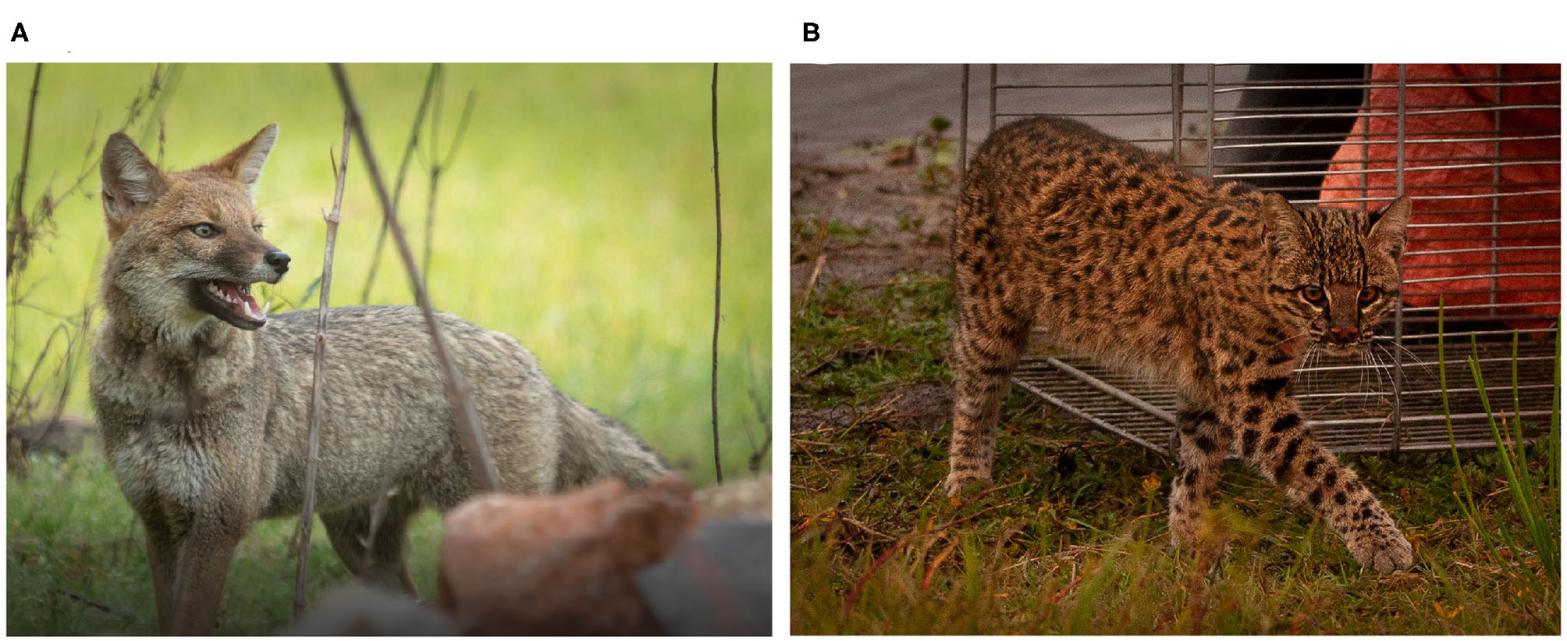
Wild Pampas fox (*Lycalopex gymnocercus*) **(A)** and Geoffroy's cat (*Leopardus geoffroyi*) **(B)** during their capture in the Brazilian Pampa Biome. *Source*: Felipe Peters.

Rectal swabs were collected from wild Pampas foxes (*n* = 5) and Geoffroy's cats (*n* = 4) (Figure 1). The animals were captured in two sites from Brazilian Pampa Biome, Rio Grande do Sul, Brazil. The first site was located near to Candiota city (31°33′06.73″S; 53°40′40.63″W), proximal to Jaguarão river, and characterized by intense agricultural, mining activity and roads; in this site, five samples were obtained. The second site was located near Arroio Grande city (32°13′58.99″S; 53°05′11.75″W), characterized by forest fragments and agricultural activities; in this site, four samples were obtained (Supplementary Table 1).

The capture, manipulation, and samples collections were authorized by Brazilian Institute of Environment and Renewable Natural Resources, IBAMA, Brasília, Brazil, and Chico Mendes Institute for Biodiversity Conservation (ICMBio). The protocol was approved by the Information Authorization System in Biodiversity (SISBIO) number 0200 1.007 9 10 12006-32. The animals were captured with the assistance of Tomahawk traps and anaesthetized via intramuscular (100 mg/mL of ketamine hydrochloride and 20 mg/mL of xylazine hydrochloride).

Rectal swabs were collected by veterinarians, all animals were clinically healthy (e.g., heart and respiratory rates and body temperature) and were classified according to gender and age group. Rectal swabs were collected from the perirectal area, stored in Stuart transport medium (Kasvi, Paraná, Brazil), and transported to our laboratory for microbiological analyses. After sample collection, the animals were returned to their habitats. All animals were in health conditions.

### Isolation and Identification of Enterococci

Isolation of enterococci was performed as described previously (17). Rectal samples were inoculated in 9 mL of azide dextrose broth (Himedia, Mumbai, India) and incubated for 24 h at 37°C. Aliquots of 1 mL were placed in 9 mL of saline water, and initial samples were further diluted 10-fold to obtain a final dilution factor of 1/1,000. From each dilution, 100 μL was inoculated in brain heart infusion (BHI) agar plates (Himedia, Mumbai, India) supplemented with 6.5% NaCl.

Since enterococci are present in high concentrations in fecal samples, typically between 10^5^ and 10^7^ CFU/g, we randomly selected 10 colonies from each fecal sample. Phenotypic criteria (size/volume, shape, color, Gram staining, catalase production), and bile esculin reaction were used to separate the enterococci group and the non-enterococcal strains. Selected pure colonies were stored at −20°C in a 10% (w/v) solution of skim milk (Difco, Sparks, MD, USA) and 10% (v/v) glycerol (Neon Comercial Ltda).

Bacterial species identification was performed by matrix-assisted laser desorption and ionization time-of-flight mass spectrometry method (MALDI-TOF) technique applied to *Enterococcus* (33). MALDI-TOF analysis was performed using a LT Bruker microflex mass spectrometer (Bruker Daltonik GmbH) and spectra were automatically identified using BrukerBioTyper™ 1.1 software. The identification by MALDI-TOF MS is based on the score value released by the equipment. A higher or similar 2.3 value indicates that the identifications of genus and species are reliable. 2.0–2.29 show that the genus is reliable and the species is probable. 1.7–1.99 values indicate that the identification of genus is probable.

### Antimicrobial Susceptibility Testing

Antimicrobial susceptibility of all strains was determined by Kirby-Bauer disk diffusion method, according to Clinical and Laboratory Standards Institute (34). Twelve antibiotics were tested: ampicillin 10 μg (AMP), vancomycin 30 μg (VAN), erythromycin 15 μg (ERY), tetracycline 30 μg (TET), ciprofloxacin 5 μg (CIP), norfloxacin 10 μg (NOR), nitrofurantoin 300 μg (NIT), chloramphenicol 30 μg (CHL), gentamicin 120 μg (GEN), linezolid 30 μg (LNZ), rifampicin 5 μg (RIF), and streptomycin 300 μg (STR). Reference strain *E. faecalis* ATCC 29212 was used as control.

Intermediate and resistant-strains were included in a single category as resistant-strains. Strains were classified as single (SR), double (DR) or multidrug-resistant (MDR) phenotype when showed resistance for one, two, and three or more antimicrobial classes, respectively (35).

### Detection of Resistance and Virulence Genes

Genomic DNA was extracted by a physicochemical method as previously described (36). The presence of resistance and virulence genes commonly observed in clinical and environmental enterococci was tested by PCR (Table 1). The resistance-related genes evaluated were: *erm*B (which encodes a ribosomal methylase that mediates macrolides, lincosamides and type B streptogramins resistance); *msr*C (which encodes for a macrolide and streptogramin B efflux pump); *tet*M and *tet*S (which encodes for tetracycline resistance via a ribosomal protection protein mechanism); and *tet*L (which encodes for tetracycline resistance via efflux pumps proteins). As well the virulence genes tested were: *ace* (adhesin to collagen of *E. faecalis*); *cyl*A (cytolysin); *agg* (aggregation substance); *gel*E (gelatinase); and *esp* (enterococcal surface protein).

**Table 1:** Primers used in the PCR reactions carried out for detection of resistance and virulence genes.

**Gene**	**Nucleotide sequence (5^**′**^-3^**′**^)**	**AT^**a**^ (**°**C)**	**Size (bp)^**b**^**	**References**
**Erythromycin**				
*erm*B_F	GAAAAGGTACTCAACCAAATA	52	645	(37)
*erm*B_R	AGTAACGGTACTTAAATTGTTTAC			
*msr*C_F	AAGGAATCCTTCTCTCTCCG	52	342	(38)
*msr*C_R	GTAAACAAAATCGTTCCCG			
**Tetracycline**				
*tet*L_F	ACTCGTAATGGTGTAGTTGC	58	627	(26)
*tet*L_R	TGTAACTCCGATGTTTAACACG			
*tet*M_F	GTTAAATAGTGTTCTTGGAG	52	656	(39)
*tet*M_R	CTAAGATATGGCTCTAACAA			
*tet*S_F	TGGAACGCCAGAGAGGTATT	58	660	(39)
*tet*S_R	ACATAGACAAGCCGTTGACC			
**Adhesion**				
*ace*_F	AAAGTAGAATTAGATCACAC	56	320	(40)
*ace*_R	TCTATCACATTCGGTTGCG			
**Cytolysin**				
*cyl*A TE17	TGGATG'ATAGTGATAGGAAGT	56	517	(41)
*cyl*A TE18	TCTACAGTAAATCTTTCGTCA			
**Biofilm**				
*esp* 46	TTACCAAGATGGTTCTGTAGGCAC	60	1198	(42)
*esp* 47	CCAAGTATACTTAGCATCTTTTGG			
**Gelatinase**				
*gel*E_F	ACCCCGTATCATTGGTTT	50	402	(41)
*gel*E_R	ACGCATTGCTTTTCCATC			
**Aggregation**				
*agg* TE3	AAGAAAAAGAAGTAGACCAAC	62	1553	(41)
*agg* TE4	AAACGGCAAGACAAGTAAATA			

a AT, annealing temperatures;

b *bp, base pair*.

Amplifications were carried out in a total volume of 25 μL containing: 100 ng of template DNA, 1 X reaction buffer (Ludwig Biotechnology), 0.4 μM of each primer (Ludwig Biotechnology), 1.5 mM MgCl_2_, 200 μM of dNTPs (Ludwig Biotechnology), 1 U Taq DNA polymerase (Ludwig Biotechnology), and MilliQ water. PCR amplifications were performed in the conventional thermocycler (Applied Biosystems 2720 Thermal Cycler) according to the following program: 94°C for 5 min followed by 35 cycles of 94°C for 1 min, appropriate annealing temperature for each primer for 1 min, extension at 72°C for 1 min, and a final extension at 72°C for 5 min. The DNA fragments amplified were analyzed in 1.5% (w/v) agarose gels stained with SYBR® Safe DNA Gel, and visualized on a photo-documenter.

## Results

In order to not overestimate the data referring to species distribution and antimicrobial susceptibility profile, strains isolated from the same animal with similar phenotypic and genotypic characteristics, which could indicate clonal strains, were grouped, generating a total of 50 strains, 30 from Pampas foxes and 20 from Geoffroy's cats. The number of isolates per wild animal ranged from 5 (samples PF3, PF4 and GC1) to 9 (sample GC3).

### Isolation and Identification of Enterococci

Enterococci were isolated from eight out of nine fecal samples. Furthermore, 50 *Enterococcus* spp. strains were isolated and characterized of wild Pampas fox and Geoffroy's cat from the Brazilian Pampa biome, including *E. faecalis* (64%; *n* = 32), *E. faecium* (22%; *n* = 11), *E. hirae* (10%; *n* = 5), and *E. durans* (4%; *n* = 2).

The species distribution between wild Pampas foxes and Geoffroy's cats are shown on Table 2. Changes in the composition of *Enterococcus* species were detected in both animals. *E. faecalis* was the most frequent species in fecal samples of both animals; however, *E. faecium* and *E. durans* were isolated only in Pampas fox and *E. hirae* just in Geoffroy's cat.

**Table 2:** Distribution of *Enterococcus* species among wild Pampas fox and Geoffroy's cat.

		**Number of species isolated**				
		***E. faecalis***	***E. faecium***	***E. hirae***	***E. durans***	**Total**
**Pampas fox**	**PF1**	4	1	0	1	6
	**PF2**	2	5	0	0	7
	**PF3**	2	3	0	0	5
	**PF4**	2	2	0	1	5
	**PF5**	7	0	0	0	7
**Geoffroy's cat**	**GC1**	5	0	0	0	5
	**GC2**	0	0	0	0	0
	**GC3**	9	0	0	0	9
	**GC4**	1	0	5	0	6
	Total	32 (64)	11 (22)	5 (10)	2 (4)	50 (100)

### Antimicrobial Susceptibility Profile

All enterococci isolated from wild canids and felids were tested for antimicrobial resistance, and almost all strains (98%, *n* = 49) were resistant to at least one evaluated antimicrobial agent (Table 3). Only one *E. hirae* isolated from Geoffroy's cat was susceptible to all antimicrobials tested. The highest frequency was found for rifampicin (94%; *n* = 47), followed by erythromycin (72%; *n* = 36), ciprofloxacin/norfloxacin (40%; *n* = 20), streptomycin (38%; *n* = 19), and tetracycline (26%; *n* = 13). Resistance to nitrofurantoin (18%; *n* = 9); gentamycin (14%, *n* = 7), and chloramphenicol (4%; *n* = 2), was noted in less frequency. No strains showed a resistance profile to ampicillin, linezolid and vancomycin.

**Table 3:** Antimicrobial resistance profiles among enterococci isolated from fecal samples of wild Pampas fox and Geoffroy's cat.

**Strains (n)**	**Number (%) of resistant strains**^****a****^								**Profiles**^****b****^		
	**ERY**	**CIP/NOR**	**RIF**	**STR**	**GEN**	**NIT**	**CHL**	**TET**	**SR**	**DR**	**MDR**
**Pampas fox**											
*E. faecalis* (17)	13 (76.47)	7 (41.18)	16 (94.12)	7 (41.18)	4 (23.53)	3 (17.65)	1 (5.88)	2 (11.76)	1 (5.88)	5 (29.41)	11 (64.70)
*E. faecium* (11)	7 (63.64)	4 (36.36)	11 (100)	4 (36.36)	0	1 (9.09)	0	4 (36.36)	1 (9.09)	4 (36.36)	6 (54.55)
*E. durans* (2)	2 (100)	0	2 (100)	1 (50)	1 (50)	0	0	1 (50)	0	0	2 (100)
Subtotal (30)	22 (73.33)	11 (36.67)	29 (96.67)	12 (40)	5 (16.67)	4 (13.33)	1 (3.33)	7 (23.33)	2 (6.67)	9 (30)	19 (63.33)
**Geoffroy's cat**											
*E. faecalis* (15)	12 (80)	9 (60)	15 (100)	3 (20)	2 (13.33)	1 (6.67)	1 (6.67)	1 (6.67)	0	4 (26.67)	10 (66.67)
*E. hirae* (5)	2 (40)	0	3 (60)	4 (80)	0	4 (80)	0	5 (100)	1 (20)	0	4 (80)
Subtotal (20)	14 (70)	9 (45)	18 (90)	7 (35)	2 (10)	5 (25)	1(5)	6 (30)	1 (5)	4 (20)	14 (70)
Total (50)	36 (72)	20 (40)	47 (94)	19 (38)	7 (14)	9 (18)	2 (4)	13 (26)	3 (6)	13 (26)	33 (66)

a *Antimicrobials: ERY, erythromycin; CIP, ciprofloxacin; NOR, norfloxacin; RIF, rifampicin; STR, streptomycin; GEN, gentamicin; NIT, nitrofurantoin; CHL, chloramphenicol; TET, tetracycline*.

b *Profiles: SR, single-resistance; DR, double-resistance; MDR, multidrug-resistance*.

The most remarkable result to emerge from the data is that a high frequency (66%; *n* = 33) of MDR strains isolated from wild canids and felids from Brazilian Pampa biome (Table 3). The percentages of double and MDR strains isolated from wild Pampas fox (30%; *n* = 9 and 63.33%; *n* = 19) were similar to wild Geoffroy's cat (20%; *n* = 4 and 70%; *n* = 14). Of the 33 MDR strains, 15 (45.45%) were resistant to four or more antimicrobials, it is important to highlight that one *E. faecalis* strain isolated from wild Pampas fox showed resistance to seven antimicrobials tested (ciprofloxacin; chloramphenicol; erythromycin; streptomycin; nitrofurantoin; rifampicin; tetracycline) (Table 4).

**Table 4:** Antimicrobial resistance phenotypic profile of *Enterococcus* sp. isolated from fecal samples of wild Pampas fox and Geoffroy's cat.

**Profile^a^**	**Antimicrobials^b^**	**Species**	**Number of resistances**	
			**PF^**c**^**	**GC^**d**^**
SR	RIF	*E. faecalis*	1	
		*E. faecium*	1	
	TET	*E. hirae*		1
DR	ERY/RIF	*E. faecalis*	3	3
		*E. faecium*	2	
	STR/RIF	*E. faecium*	1	
	CIP-NOR/RIF	*E. faecalis*	1	1
		*E. faecium*	1	
	NIT/RIF	*E. faecalis*	1	
MDR	CIP-NOR/ERY/RIF	*E. faecalis*	3	4
		*E. faecium*	1	
	CIP/STR/RIF	*E. faecalis*	1	
	CIP/ERY/TET	*E. faecium*	1	
	CIP/CHL/RIF	*E. faecalis*		1
	ERY/STR/TET	*E.durans*	1	
	ERY/GEN/RIF	*E. faecalis*	1	
		*E. durans*	1	
	ERY/STR/RIF	*E. faecium*	1	
	STR/GEN/RIF	*E. faecalis*	1	
	CHL/ERY/RIF	*E. faecalis*		1
	CIP/ERY/GEN/RIF	*E. faecalis*		1
	CIP/STR/GEN/RIF	*E. faecalis*		2
	CIP/ERY/STR/RIF	*E. faecalis*	1	1
	STR/NIT/TET/NOR	*E. hirae*		1
	STR/NIT/TET/RIF	*E. hirae*		1
	ERY/STR/GEN/RIF	*E. faecalis*	1	
	ERY/STR/TET/RIF	*E. faecium*	1	
	ERY/STR/NIT/TET/RIF	*E. faecium*	1	
		*E. faecalis*	1	1
		*E. hirae*		2
	CIP/ERY/STR/GEN/RIF	*E. faecalis*	1	
	CIP/CHL/ERY/STR/NIT/TET/RIF	*E. faecalis*	1	

a *SR, single-resistance; DR, double-resistance; MDR, multidrug-resistance*.

b *Antimicrobials: ERY, erythromycin; CIP, ciprofloxacin; NOR, norfloxacin; RIF, rifampicin; STR, streptomycin; GEN, gentamicin; NIT, nitrofurantoin; CHL, chloramphenicol; TET, tetracycline*.

c *PF, Pampas fox (L. gymnocercus)*.

d *GC, Geoffroy's cat (L. geoffroyi)*.

### Frequency of Antimicrobial Resistance and Virulence Related Genes

The resistance genes were investigated only in phenotypically resistant erythromycin and tetracycline strains (Table 5). Of the 36 erythromycin- resistant, four (11.11%) harbored *erm*B and nine (25%) *msr*C genes. Among the 13 tetracycline-resistant enterococci, *tet*L and *tet*M genes were found in 7 (53.85%) strains. None strain was positive to *tet*S gene.

**Table 5:** Distribution of erythromycin- and tetracycline-resistance genes in the enterococci isolated from wild Pampas Fox and Geoffroy's cat.

	**Strains**	**Number (%) of strains positive for resistance genes**						
		**Erythromycin**			**Tetracycline**			
		R^*^	***erm*B**	***msr*C**	R^*^	***tet*M**	***tet*L**	***tet*S**
Pampa fox	*E. faecalis*	13	0	5 (38.46)	2	0	0	0
	*E. faecium*	7	0	3 (42.86)	4	0	0	0
	*E. durans*	2	1 (50)	1 (50)	1	1 (100)	1 (100)	0
	Subtotal	22	1 (4.55)	9 (40.91)	7	1 (14.29)	1 (14.29)	0
Geoffroy's cat	*E. faecalis*	12	1 (8.33)	0	1	1 (100)	1 (100)	0
	*E. hirae*	2	2 (100)	0	5	5 (100)	5 (100)	0
	Subtotal	14	3 (21.43)	0	6	6 (100)	6 (100)	0
	Total	36	4 (11.11)	9 (25)	13	7 (53.85)	7 (53.85)	0

* *Resistant strains*.

All strains were tested for the presence of enterococci commonly associated virulence genes. The Table 6 shows the results of *gelE, cylA, esp, ace*, and *agg* genes. The highest frequencies of virulence genes were found in *E. faecalis* and *E. faecium*. The *gelE* (62%; *n* = 31) and ace (48%; *n* = 24) showed elevated prevalence among these species. The *agg* gene (22%; *n* = 11) was recorded only on *E. faecalis* strains. Otherwise, *esp* and *cylA* genes were observed in just one *E. faecium* and *E. hirae* strains, respectively.

**Table 6:** Number (%) of virulence genes among enterococci isolated from wild Pampas Foxes and Geoffroy's cat.

	**Pampas fox**			**Geoffroy's cat**		
**Virulence genes**	***E. faecalis* (*n* = 17)**	***E. faecium* (*n* = 11)**	***E. durans* (*n* = 2)**	***E. faecalis* (*n* = 15)**	***E. hirae* (*n* = 5)**	**Total (%)**
*gel*E	12 (70.59)	5 (45.45)	0	14 (93.33)	0	31 (62)
*cyl*A	0	0	0	0	1 (20)	1 (2)
*esp*	0	1 (9.09)	0	0	0	1 (2)
*ace*	12 (70.59)	7 (63.64)	0	5 (33.33)	0	24 (48)
*agg*	7 (41.18)	0	0	4 (26.67)	0	11 (22)

## Discussion

### Isolation and Identification of Enterococci

Relatively few studies have reported enterococci isolated from wild canids and felids such as red foxes (43), Iberian wolves, and Iberian lynx (44, 45). The results of the present study corroborate with previous results showing that *E. faecalis, E. faecium, E. hirae*, and *E. durans* are commonly encountered in the fecal samples of wild and domestic canids and felids (31, 43–47). However, when we verified the distribution of enterococci in Pampas foxes and Geoffroy's cats, we observed a higher frequency of *E. faecalis* than those previously reported for wild red foxes, Iberian lynx, and Iberian wolves (44, 45). Moreover, our results are comparable to those of domestic canids and felids (31, 46, 47) since frequencies of *E. faecalis* (64.9%), *E. faecium* (18.2%), and *E. durans* (6.5%) were detected. This minor disagreement is supported by the fact that the distribution of enterococci may vary according to individual characteristics (e.g., species, age, and sex), habitat (e.g., seasonal variations and diet), and the geographic distribution of the animals (20).

Enterococcal species prevalence varied according to the host species studied. Although these species occupy the same area of the Biome, several types of foods are available to them. Geoffroy's cat and Pampas fox are considered generalist omnivores that opportunistically feed on a wide variety of foods. Pampas fox has a diet dominated by animal prey, mainly wild mammals, insects, while the Geoffroy cat feeds mainly on rodents and hares, and also remains of fish and frogs alongside reptiles and birds (48, 49). Thus, the distribution of *Enterococcus* species among hosts observed in the present study can be justified by the availability of the animals' food, since enterococcal species have been isolated from mammals, birds, fish, insects, and reptiles (20).

Notably, it was not possible to isolate enterococci from one of Geoffroy's cat fecal samples. Previously, Santestevan et al. (50) and Layton et al. (51) also sought to isolate enterococci from mammalian fecal samples and were unsuccessful.

### Antimicrobial Susceptibility Profile

The results of this study are consistent with previous studies, which found high rates of resistance to erythromycin (65%), ciprofloxacin (59.5%), and tetracycline (36.5%) in fecal enterococci isolates from wild mammals, including wolves and foxes (31). Some reports have detected enterococci resistant to tetracycline and erythromycin in wild Iberian wolves, Iberian lynx, and red foxes in Portugal (43–45). Additionally, domestic canids and felids also harbored antimicrobial-resistant enterococci (47, 52, 53).

While MDR enterococci strains have previously been observed in enterococci isolated from wild mammals, their resistance levels were not as high as those detected here. In the present study, 66% of MDR was observed for wild canids and felids from the Brazilian Pampa biome. The high frequency of MDR strains may be associated with the proximity of these animals to human activities since they are sentinel species (i.e., indicators of danger to the environment). It is commonly known that wild canids and felids are indifferent to the presence of humans and often share the same environment. Our results are in line with those of Nowakiewicz et al. (54), who observed a high frequency of *E. faecalis* strains (44%) among wild mammalian carnivores in Poland. On the other hand, our data are six times higher than those detected by Dec et al. (30). According to Hu et al. (55), MDR bacteria are more commonly associated with environmental contamination than naturally occurring genes. Moreover, studies of wild foxes and carnivorous mammals revealed positive correlations with environmental pollution and the abundance of resistant bacteria in samples, thereby highlighting the selective pressures that human activities and environmental disturbances exert on the microbial communities of wildlife (31, 54).

The elevated frequency of resistant and MDR enterococci observed in the fecal samples of wild Pampas foxes and Geoffroy's cats might be associated with anthropogenic activities. Agriculture and livestock are the main economic activities in the Brazilian Pampa and represents a source of food for billions of people and animals (mainly cattle and sheep). Since 1998, many drugs have been prohibited from being used as growth promoters in Brazil. In livestock, antimicrobials such as amoxicillin, erythromycin and tetracycline are used by veterinarians to treat bacterial infections (56). Despite bringing benefits to production, the use of antimicrobials in animals has fostered the emergence and spread of antimicrobial resistance. Antibiotics and/or antibiotic-resistant bacteria can be secreted with animal urine and feces and contaminate the environments (soils, surface waters, and ground waters) and species inhabiting these environments (57). In the presence of environmental concentrations of antibiotics, bacteria face a selective pressure leading to a gradual increase in the prevalence of resistance. The association of antibiotic resistance genes in mobile genetic elements is also an important factor for spreading and persistence of antimicrobial resistance in the environment (58). It is important to highlight that the impact created by the presence of antimicrobial agents in the environment and the frequency with which these resistance genes are transferred remains a subject of academic and practical debate. Our results suggest that the impacted environment occupied by Pampas foxes and Geoffroy's cats —with intense agricultural and livestock activities in the sampling area—possibly contributed to the selection of resistant bacteria in the environment and subsequent acquisition of resistant strains by these mammals. Despite anthropogenic activities, the presence of antibiotic-resistant strains in wild animals may also be associated with the environmental resistome, which is composed of genes that naturally occur in the environment (59). One example is the genes associated with the expression of efflux pumps, which protect cells against toxic molecules such as heavy metals, expelling them to the external environment and leading to antimicrobial resistance (60).

### Frequency of Antibiotic Resistance Genes

The *erm*B and *msr*C genes, conferring resistance to macrolides, were present in 11.11 and 25% of isolates, respectively. The low frequency of *erm*B genes detected in the present study is congruent with the results obtained in previous studies conducted on *Enterococcus* strains isolated from wild animals (17, 18, 30, 50), as in regarding to *msr*C gene (28). Additionally, we detected the presence of the *msr*C gene not only in *E. faecium* but also in *E. durans* and *E. faecalis*. Although the *msr*C gene is considered an intrinsic gene to *E. faecium*, some studies have noted the presence of this gene in other *Enterococcus* species such as *E. hirae* and *E. faecalis* (30, 38).

In the present study, *tet*L and *tet*M genes were detected in tetracycline-resistant enterococci strains. Previous findings of enterococci in wild animals such as Iberian wolves and Iberian lynx also harbored those genes in tetracycline-resistant strains (44, 45). Some erythromycin- and tetracycline-resistant strains did not amplify for the tested gene and may carry other antibiotic resistance genes such as *erm*A, C, D, E, F, G, Q, *msr*A/B, other *tet*-group genes, and the *poxt*A gene for tetracycline-resistance (61). Our results point to the notion that other reported genes could be associated with erythromycin-resistant enterococci isolated from Pampas foxes and Geoffroy's cats. Furthermore, whole-genome sequencing (WGS) of these enterococci might be useful in identifying additional mechanisms associated with resistance profiles.

Antibiotic resistance genes commonly reside on transmissible plasmids or on other mobile genetic elements, which allow the horizontal transfer of these genes between strains. The *tet*M, *tet*L, and *erm*B genes are carried out by mobile genetic elements, such as transposons (Tn*916*, Tn*1545*, and Tn*917*), conjugative transposons or plasmids (58). The association of these genes in mobile genetic elements might be an important factor for spreading of antimicrobial resistant enterococci in wild Pampas foxes and Geoffroy's cats.

### Frequency of Virulence-Related Genes

The results of the present study suggest that enterococci obtained from wild Pampas foxes and Geoffroy's cats harbored virulence genes. Moreover, *E. faecalis* was the most common species to carry virulence factors. These results are congruent with previous studies highlighting *E. faecalis* as the most common enterococcal species associated with infections, which accounts for 80–90% of infections. The presence of virulence factors in clinical enterococci strains is associated with persistent and difficult-to-treat infections. However, some authors consider the occurrence of these genes in non-clinical strains as a common characteristic that increases their ability to colonize hosts, which improves the survival and proliferation of the strains. Since the ubiquity of enterococci across a wide range of environments was initiated by the establishment of these bacteria in either abiotic surfaces or live tissues, their colonization can be facilitated by the expression of virulence genes that likely contribute to the persistence of enterococci in the environment (20).

One limitation of our study is the low number of animals sampled, which is due to the difficulty of obtaining samples from wildlife. For example, a study conducted in an anthropogenic area of the Brazilian Pampa during a 1 year period, 12 Geoffroy's cat individuals were captured (62). Notably, capturing and handling wild animals requires specialized equipment, the consideration of animal welfare concerns (regardless of the reason for capture), and the efforts of experienced biologists and wildlife technicians to plan and study suitable capture methods. In light of these points, the number of animals evaluated in the present study should be well-considered. Despite its relatively small sample size, this study demonstrated the importance of conducting research related to the impact of human activities on the Brazilian Pampa biome.

In conclusion, this study observed the presence of resistant *Enterococcus* strains in wild Pampas foxes and Geoffroy's cats from the Brazilian Pampa biome. The presence of MDR enterococci in fecal samples from these wild animals suggests that habitat fragmentation and the impact of anthropogenic activities on the environment might contribute to the occurrence of resistant strains in the microbial gut communities of these animals. Furthermore, these animals may contribute to the spread of resistant strains between different ecosystems. To the best of our knowledge, this is the first study of resistant commensal enterococci recovered from wild animals in the Brazilian Pampa biome. We believe that our research will serve as a foundation for future studies on the Pampa biome.

## Data Availability Statement

The original contributions presented in the study are included in the article/Supplementary Materials, further inquiries can be directed to the corresponding author.

## Ethics Statement

The animal study was reviewed and approved by Instituto Brasileiro do Meio Ambiente e dos Recursos Naturais Renováveis (IBAMA), and Chico Mendes Institute for Biodiversity Conservation (ICMBio). The protocol was approved by Information Authorization System in Biodiversity (SISBIO) no. 0200 1.007 9 10 12006-32.

## Author Contributions

GO, JF, and AG designed the study. FP and MF carried out the sampling work. GO, RH, MM, JF, and AG analyzed the data and drafted the manuscript. All authors have read and approved the final manuscript.

## Conflict of Interest

The authors declare that the research was conducted in the absence of any commercial or financial relationships that could be construed as a potential conflict of interest.
