# Supplementary material for: Multidrug Resistance in Enterococci Isolated From Wild Pampas Foxes (Lycalopex gymnocercus) and Geoffroy's Cats (Leopardus geoffroyi) in the Brazilian Pampa Biome
Source: Front Vet Sci. 2020 Dec 4;7:606377. doi: 10.3389/fvets.2020.606377 (PMC7793794; doi:10.3389/fvets.2020.606377)
Supplement: Supplementary file 1 [file Table_1.DOCX]

Supplementary Material

# Supplementary Table 1. Details of wild Pampas fox (*Lycalopex gymnocercus*) and Geoffroy’s cat (*Leopardus geoffroyi*) identification and collection sites of Brazilian Pampa biome.

| **Specie** | **Animal identification¹** | **Sex²** | **Collection site** |
| --- | --- | --- | --- |
| **Pampas fox**  **(*L. gymnocercus*)** | PF1 | M | Candiota |
|  | PF2 | F | Candiota |
|  | PF3 | M | Candiota |
|  | PF4 | M | Candiota |
|  | PF5 | M | Arroio Grande |
| **Greoffroy’s cat**  **(*L. geoffroyi*)** | GC1 | F | Candiota |
|  | GC2 | F | Arroio Grande |
|  | GC3 | M | Arroio Grande |
|  | GC4 | M | Arroio Grande |

¹: PF, Pampas fox; GC, Geoffroy’s cat. ²: F, female; M, male.
